# Ursa: A Comprehensive Multiomics Toolbox for High-Throughput Single-Cell Analysis

**DOI:** 10.1093/molbev/msad267

**Published:** 2023-12-13

**Authors:** Lu Pan, Tian Mou, Yue Huang, Weifeng Hong, Min Yu, Xuexin Li

**Affiliations:** Institute of Environmental Medicine, Karolinska Institutet, Solna 171 65, Sweden; Department of Medical Epidemiology and Biostatistics, Karolinska Institutet, Solna 171 65, Sweden; School of Biomedical Engineering, Shenzhen University, Shenzhen, Guangdong 518060, China; Department of Medical Epidemiology and Biostatistics, Karolinska Institutet, Solna 171 65, Sweden; Department of Radiation Oncology, Zhongshan Hospital, Fudan University, Shanghai 200032, China; Department of General Surgery, Guangdong Provincial People’s Hospital (Guangdong Academy of Medical Sciences), Southern Medical University, Guangzhou, Guangdong 510515, China; Department of Medical Biochemistry and Biophysics, Karolinska Institutet, Solna 171 65, Sweden; Department of General Surgery, The Fourth Affiliated Hospital, China Medical University, Shenyang 110032, China

**Keywords:** multiomics, single-cell, analysis workflow, multimodal analysis

## Abstract

The burgeoning amount of single-cell data has been accompanied by revolutionary changes to computational methods to map, quantify, and analyze the outputs of these cutting-edge technologies. Many are still unable to reap the benefits of these advancements due to the lack of bioinformatics expertise. To address this issue, we present Ursa, an automated single-cell multiomics R package containing 6 automated single-cell omics and spatial transcriptomics workflows. Ursa allows scientists to carry out post-quantification single or multiomics analyses in genomics, transcriptomics, epigenetics, proteomics, and immunomics at the single-cell level. It serves as a 1-stop analytic solution by providing users with outcomes to quality control assessments, multidimensional analyses such as dimension reduction and clustering, and extended analyses such as pseudotime trajectory and gene-set enrichment analyses. Ursa aims bridge the gap between those with bioinformatics expertise and those without by providing an easy-to-use bioinformatics package for scientists in hoping to accelerate their research potential. Ursa is freely available at https://github.com/singlecellomics/ursa.

## Introduction

Single-cell technologies have equipped us with the ability to observe cellular events explicitly and multi-dimensionally. The emergence of these new omics technologies not only leveraged the perspective of observing biological events at the single-cell level but also created opportunities for researchers to observe different molecular layers in molecular complexity and cell population variation simultaneously. The immense potential of single-cell technologies to unveil novel insights has demonstrated remarkable achievements after successive efforts of scientists in making new discoveries at the single-cell level (Lawson et al. 2018; Suvà and Tirosh 2019; Aldridge and Teichmann 2020; Ginhoux et al. 2022). Ever since the advent of the first single-cell technology back in 2009 (Tang et al. 2009), there has been an apparent outburst of single-cell studies across multiple omics over the past few decades (Amezquita et al. 2020; Li et al. 2022). The influx of multidimensional data requires biology to be more and more computationally dependent, which has now transformed biology into computational biology that brings even more challenges to scientists, especially to clinicians or bench scientists who are computationally less competent. To date, big data complexity and data analysis methods have undergone revolutionary changes in data volume and their dimension and interpretation (Hwang et al. 2018; Stuart and Satija 2019; Amezquita et al. 2020; Kashima et al. 2020; Lee et al. 2020; Ma et al. 2020) and are accompanied by incremental growth in the formulation of new analysis methods and computational tools for the interpretation of big data (Gardeux et al. 2017; Hwang et al. 2018; Stuart and Satija 2019; Amezquita et al. 2020; Franzén and Björkegren 2020; Kashima et al. 2020; Lee et al. 2020; Ma et al. 2020; Taverna et al. 2020; Yousif et al. 2020; Moreno et al. 2021; Pereira et al. 2021; Zappia and Theis 2021; Hasanaj et al. 2022; Jiang et al. 2022; Prieto et al. 2022; Wang et al. 2023; Chen et al. 2023). Currently, the majority of the bioinformatics tools available are predominantly designed for single-cell RNA-sequencing (scRNA-Seq) data analysis (Zappia and Theis 2021), and fewer tools are tailored to other omics types such as scATAC-Seq (Stuart et al. 2021) and spatial transcriptomics (Kleino et al. 2022). The complexity of these tools often necessitates multiple coding procedures, effectively limiting their use to computational scientists. This creates a steep learning curve for those with limited computational skills. Consequently, clinicians and bench scientists, who could greatly benefit from these tools, are often sidelined. The growing gap between “computationally skilled” and “computationally less competent” scientists is exacerbated by the rapid development of single-cell technologies and methods. This disparity poses a significant barrier, hindering the effective utilization of these innovative technologies in broader scientific research.

Hereby, we present an effort in unifying promising single-cell and spatial transcriptomics analyses and algorithms, transforming them into automated single-cell omics and spatial transcriptomics workflows, and compiling them into a 1-stop solution named Ursa. Ursa is the first ever single-cell multiomics software to include almost all single-cell omics analysis workflows. Internal tools for every part of the analysis were chosen based on their performance and runtime, to run as little codes as possible, i.e. 1-liner command for each omics analysis, and at the same time, to cover as many analysis procedures as possible. Popular tools and vibrant set of downstream analysis, including pseudotime trajectory analysis, and gene-set enrichment analysis, could be done easily with just 1 command. In detail, Ursa consists of automated analysis workflows of 6 single-cell omics and 1 bulk omics, including scRNA-Seq, scATAC-Seq, spatial transcriptomics, single-cell copy number variations (scCNV), scImmune profiling, CyTOF, and flow cytometry, and is compatible with a wide array of technological outputs. It also supports multimodal integrative analysis such as integrating scRNA-Seq data with scImmune profiling data. It is the first ever single-cell multiomics tool capable of handling complex workflows across 6 different omics.

## Results

### Current Trend in Single-Cell Omics Tools

**Fig. 1.**
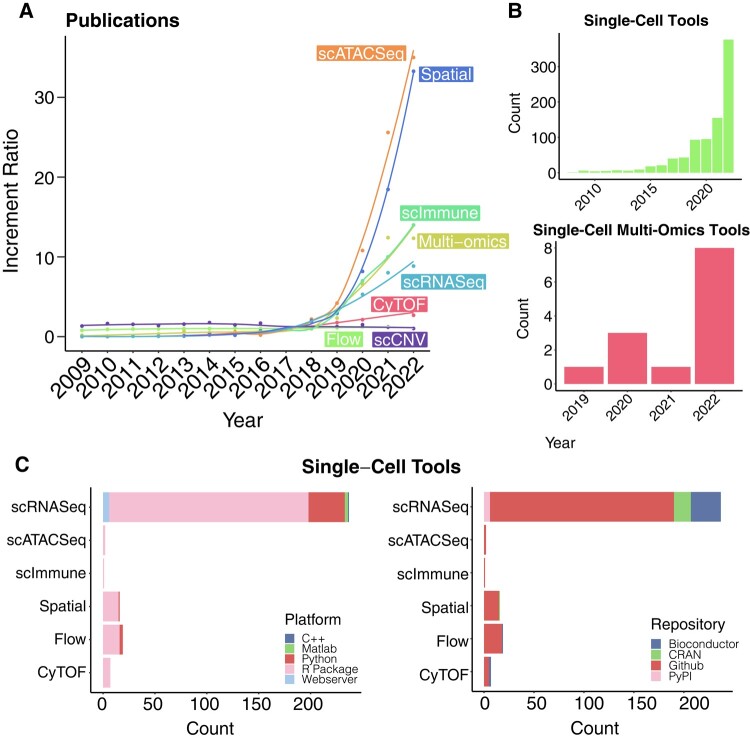
Trends of single-cell omics and their tools the past decades. (A) Increment ratio of the number of publications from PubMed since 2009 in natural scale. Ratio represents the number of omics publications with reference to the number of publications in each omics in year 2017 (the year for the latest omics technology emergence across comparing omics). Curves indicated the fitted increment ratio for each omics. (B) Number of published single-cell tools and single-cell multiomics tools over the past decades. (C) Availability of tools across different programming languages (left), and across different data repositories.

We conducted an extensive review on the publications of single-cell omics studies from the year 2009 (i.e. the year of the emergence of the first single-cell technology) up to year 2022, summarizing single-cell omics studies and tools (Fig. 1). Our search strategy included the short abbreviations and full terms of each omics, with variations accounting for the presence and absence of intermediate symbols such as dash. As shown in Fig. 1a, many single-cell omics studies exhibited a marked exponential growth, notably in scATAC-Seq, spatial transcriptomics, scImmune profiling, and scRNA-Seq. Concurrently, multiomics studies are also on the rise. This surge is paralleled by a rise in the development of analysis methods for these omics types. As Fig. 1b shows, while the number of tools for single-cell omics has escalated, multiomics tools are comparatively fewer. Most existing tools primarily catered to scRNA-Seq (Fig. 1c), leaving other rapidly emerging omics types less addressed. Given the substantial growth in scATAC-Seq and other omics, as well as multiomics research, there is a pressing need to develop user-friendly, versatile multiomics tools that can integrate various single-cell omics data.

### Comparison Between Single-Cell Tools With Minimal or No Programming

To broaden user accessibility, we developed Ursa, which is a computationally less intensive tool for single-cell multiomics analyses. We compared Ursa against a selection of tools known for their minimal programming complexity (Gardeux et al. 2017; Hwang et al. 2018; Stuart and Satija 2019; Amezquita et al. 2020; Franzén and Björkegren 2020; Kashima et al. 2020; Lee et al. 2020; Ma et al. 2020; Taverna et al. 2020; Yousif et al. 2020; Moreno et al. 2021; Pereira et al. 2021; Zappia and Theis 2021; Hasanaj et al. 2022; Jiang et al. 2022; Prieto et al. 2022; Wang et al. 2023; Chen et al. 2023) (Table 1). It is evident that many of these tools possess a relatively constrained range in their analytic capabilities. Tools such as Asc-Seurat (Pereira et al. 2021), Alona (Franzén and Björkegren 2020), SCTK (Wang et al. 2023), NASQAR (Yousif et al. 2020), and ICARUS (Jiang et al. 2022) primarily focus on analytic workflows tailored for scRNA-Seq technologies (Table 1). Conversely, platforms such as Partek Flow, Qiagen, ezSingleCell (Chen et al. 2023), and Cellar (Hasanaj et al. 2022) have expanded their range to encompass up to 3 single-cell omics, including scRNA-Seq, scATAC-Seq, and either spatial transcriptomics or scImmune profiling. In contrast, Ursa stands out by encompassing a broader range of single-cell omics types, while simultaneously simplifying analysis procedures to be more accessible for users with limited computational skills. This approach not only expands the utility of Ursa but also makes it a valuable tool for a wider range of researchers, especially those less versed in computational techniques.

**Table 1:** Comparison of Ursa with other advanced single-cell multiomics tools

Feature/software	Ursa	Partek Flow	QIAGEN	ezSingleCell	BIOMEX	Cellar	Asc-Seurat	Alona	SCTK	NASQAR	ICARUS	SCiAp
*Omics types (single-cell level)*												
scRNA-Seq	✅	✅	✅	✅	✅	✅	✅	✅	✅	✅	✅	✅
scATAC-Seq	✅	✅	✅	✅	✖	✅	✖	✖	✖	✖	✖	✅
scImmune profiling	✅	✗	✅	✖	✖	✖	✖	✖	✖	✖	✖	✖
scCNV	✅	✖	✖	✖	✖	✖	✖	✖	✖	✖	✖	✖
Spatial transcriptomics	✅	✅	✖	✅	✖	✅	✖	✖	✖	✖	✖	✖
CyTOF	✅	✖	✖	✖	✅	✖	✖	✖	✖	✖	✖	✖
Flow cytometry	✅	✖	✖	✖	✖	✖	✖	✖	✖	✖	✖	✖
*Availability*												
Open-source	✅	✖	✖	✅	✅	✅	✅	✅	✅	✅	✅	✅
Free of charge/noncommercial	✅	✖	✖	✅	✅	✅	✅	✅	✅	✅	✅	✅
*Complexity*												
Coding												
Minimum coding/no coding	✅	✅	✅	✅	✅	✅	✅	✅	✅	✅	✅	✅
*User-interface (UI)*												
Interactive UI with adjustable features	✖	✅	✅	✅	✅	✅	✅	✅	✅	✅	✅	✅
*Workflow*												
scRNA-Seq												
Quality control	✅	✅	✅	✅	✅	✅	✅	✅	✅	✅	✅	✅
Data processing	✅	✅	✅	✅	✅	✅	✅	✅	✅	✅	✅	✅
Phenotypic discovery and statistical analysis	✅	✅	✅	✅	✅	✅	✅	✅	✅	✅	✅	✅
Integrative analysis	✅	✅	✅	✅	✅	✅	✅	✅	✅	✖	✅	✅
scATAC-Seq												
Quality control	✅	✅	✅	✅	✖	✅	✖	✖	✖	✖	✖	✅
Data processing	✅		✅	✅	✖	✅	✖	✖	✖	✖	✖	✖
Phenotypic discovery and statistical analysis	✅	✅	✅	✅	✖	✅	✖	✖	✖	✖	✖	✖
Integrative analysis	✅	✖	✖	✖	✖	✅	✖	✖	✖	✖	✖	✖
Multimodal analysis	✅	✖	✖	✅	✖	✅	✖	✖	✖	✖	✖	✖
scImmune profiling												
Phenotypic assessments	✅	✖	✅	✖	✖	✖	✖	✖	✖	✖	✖	✖
Multimodal analysis	✅	✖	✅	✖	✖	✖	✖	✖	✖	✖	✖	✖
scCNV												
Quality control	✅	✖	✖	✖	✖	✖	✖	✖	✖	✖	✖	✖
Data processing	✅	✖	✖	✖	✖	✖	✖	✖	✖	✖	✖	✖
Phenotypic discovery and statistical analysis	✅	✖	✖	✖	✖	✖	✖	✖	✖	✖	✖	✖
Spatial transcriptomics												
Quality control	✅	✅	✖	✅	✖	✅	✖	✖	✖	✖	✖	✖
Data processing	✅	✅	✖	✅	✖	✅	✖	✖	✖	✖	✖	✖
Phenotypic discovery and statistical analysis	✅	✅	✖	✅	✖	✅	✖	✖	✖	✖	✖	✖
Multimodal analysis	✅	✖	✖	✅	✖	✅	✖	✖	✖	✖	✖	✖
CyTOF												
Data processing	✅	✖	✖	✖	✅	✖	✖	✖	✖	✖	✖	✖
Phenotypic discovery and statistical analysis	✅	✖	✖	✖	✅	✖	✖	✖	✖	✖	✖	✖
Flow cytometry												
Quality control	✅	✖	✖	✖	✖	✖	✖	✖	✖	✖	✖	✖
Auto-gating	✅	✖	✖	✖	✖	✖	✖	✖	✖	✖	✖	✖
Data processing	✅	✖	✖	✖	✖	✖	✖	✖	✖	✖	✖	✖
Phenotypic discovery and statistical analysis	✅	✖	✖	✖	✖	✖	✖	✖	✖	✖	✖	✖
*Acceptable technologies*												
scRNA-Seq												
10X Genomics	✅	✅	✅	✅	✅	✅	✅	✅	✅	✅	✅	✅
Multimodal 10X Genomics	✅	✅	✅	✅	✅	✅	✅	✅	✅	✅	✅	✅
Smart-Seq2	✅	✅	✅	✅	✅	✅	✖	✅	✅	✅	✅	✅
CITE-Seq	✅	✅	✅	✅	✅	✅	✖	✅	✅	✅	✅	✅
Dropseq	✅	✅	✅	✅	✅	✅	✖	✅	✅	✅	✅	✅
scATAC-Seq												
10X Genomics	✅	✖	✖	✅	✖	✅	✖	✖	✖	✖	✖	✅
Multimodal 10X Genomics	✅	✖	✖	✅	✖	✅	✖	✖	✖	✖	✖	✅
scImmune profiling												
10X Genomics	✅	✖	✖	✖	✖	✖	✖	✖	✖	✖	✖	✖
Multimodal 10X Genomics	✅	✖	✖	✖	✖	✖	✖	✖	✖	✖	✖	✖
scCNV												
10X Genomics	✅	✖	✖	✖	✖	✖	✖	✖	✖	✖	✖	✖
Spatial transcriptomics												
10X Genomics Visium	✅	✅	✖	✅	✖	✅	✖	✖	✖	✖	✖	✖
Multimodal 10X Genomics	✅	✅	✖	✅	✖	✅	✖	✖	✖	✖	✖	✖
CyTOF												
CyTOF Helios v1,v2	✅	✖	✖	✖	✅	✖	✖	✖	✖	✖	✖	✖
Flow cytometry												
Support various flow cytometry technologies	✅	✖	✖	✖	✖	✖	✖	✖	✖	✖	✖	✖

Tick indicates the availability of the specific feature, while a cross indicates its absence.

### Ursa: A Single-Cell Multiomics Software

**Fig. 2.**
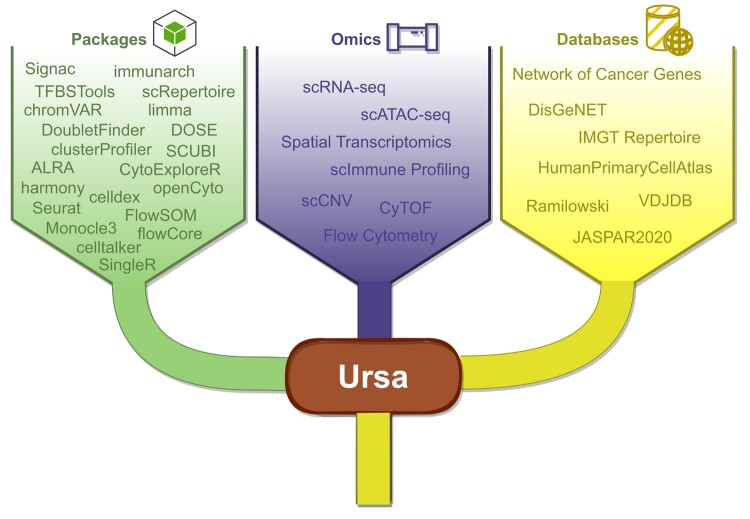
Lists of main analytic tools and databases used by Ursa. Diagram summarizes (from left to right) a list of major packages used by Ursa, the number of omics pipelines included in Ursa, and a list of databases used by Ursa.

**Fig. 3.**
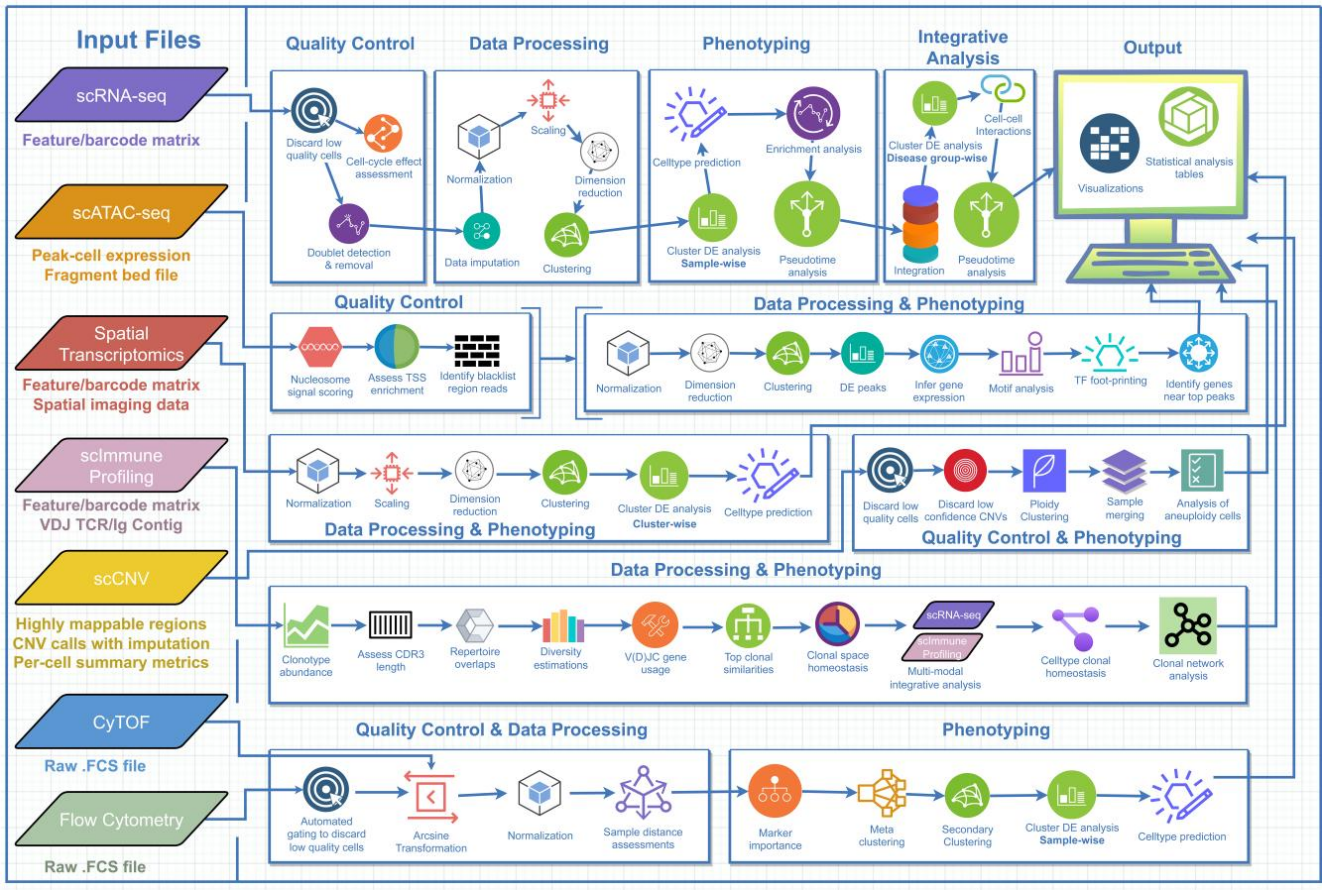
An overview of Ursa analysis workflows. Ursa accepts input files from scRNA-Seq, scATAC-Seq, spatial transcriptomics, scImmune profiling, scCNV, CyTOF, and flow cytometry data to carry out bioinformatics analysis for each of these omics. For scRNA-Seq multiple samples project, integrative analysis will be carried out in addition to sample-wise analysis. For flow cytometry, automated gating is also provided to replace manual gating out of low-quality cells.

Ursa encompassed up to 6 single-cell omics and spatial transcriptomics, which is capable of handling post-quantification data from an array of technologies across these omics (Table 1). Utilizing a combination of established methods and databases (Fig. 2, supplementary table S1, Supplementary Material online), Ursa emerged as the first single-cell multiomics tool offering a set of comprehensive analytic workflows. The workflows spans from quality control all the way to phenotypic discoveries (Fig. 3, Table 1). Moreover, Ursa also supports multimodal integrative analysis such as integrating scRNA-Seq data with scImmune profiling data, offering insights unattainable through single-modal analysis. In the current landscape of single-cell omics tools, none of the available single-cell omics tools offer the breadth found in Ursa. Ursa stands unparalleled in its range, being the sole single-cell multiomics tool that delivers a comprehensive suite of both single and multimodal omics analytical workflows, encompassing 6 single-cell omics modalities along with spatial transcriptomics while being open-source and freely available.

### Ursa Implementation

To run, the user is required to provide in the input directory, direct post-quantification output files (as stated in the Materials and Methods) and a *.txt* or *.csv* meta file containing the file name, sample id, batch as well as group information for each sample of the project. A 1-liner command for each omics will be passed to R and will trigger a cascade of analysis processes automatically (Fig. 3,supplementary table S2). Users are free to adjust for parameters such as the percentage of mitochondrial reads to filter for by adding the parameter name and value in the 1-liner command. If the parameters are not indicated by the user, default settings will be automatically implemented. A subfolder named after the project name provided by the user, concatenated with a timestamp, will be created in the user-stated output directory and all output files or plots from the current project will be deposited inside. At most of the steps in all analysis workflows, plots will be generated to facilitate the understanding of each step, as well as to increase plot choices for users to use in their publications. The Ursa software is also capable of supporting large-scale on-server analyses and is compatible with Linux, Windows, and OS X. To install Ursa in the R environment, please refer to the instruction page on Github (https://github.com/singlecellomics/ursa).

#### scRNA-Seq

For scRNA-Seq, user is required to provide in the input directory, direct 10X Genomics Cellranger (Weisenfeld et al. 2017; Zheng et al. 2017) *.h5* format sample output files, or gene-to-cell matrix files in *.txt* or *.csv* format from other technologies such as Smart-Seq2 (Picelli et al. 2014), CITE-Seq (Stoeckius et al. 2017), or Dropseq (Macosko Evan et al. 2015). A *.txt* or *.csv* meta file containing file name, sample id, batch as well as group information for each sample is needed from the user. To start the analysis, user is required to run the following command in an R environment after installing and loading Ursa:

scRNASEQPip(project_name = ‘My_scRNASeq’, input_dir = ‘/home/input/’, output_dir = ‘/home/output/’, pheno_file = ‘/home/input/meta.txt’)

Other parameters can be added to the command, and default settings and the origin of the parameters from their source dependency packages can be found in supplementary table S2, Supplementary Material online or under the Help page of each command in the R environment; likewise for the commands to run all other omics. For example, to change the default setting of the quality control step for filtering out cells based on their percentage of mitochondrial reads, the parameter *seurat.max.mito.percent* and its value can be added to the above command, such as, if the user would like to change from the default setting of filtering out cells with more than 10% mitochondrial reads to 20%, the above command can be changed to the following before running:

scRNASEQPip(project_name = ‘My_scRNASeq’, input_dir = ‘/home/input/’, output_dir = ‘/home/output/’, pheno_file = ‘/home/input/meta.txt’, seurat.max.mito.percent = 20)

#### scATAC-Seq

For scATAC-Seq, user is required to provide in the input directory, post-Cellranger scATAC-Seq files. Meta file should contain sufficient information to locate sample file, sample id, batch as well as group information for each sample. To start the analysis, user is required to run the following command:

scATACPip(project_name = ‘My_scATAC’, input_dir = ‘/home/input/’, output_dir = ‘/home/output/’, pheno_file = ‘/home/input/meta.txt’)

#### scImmune Profiling

For scImmune profiling, user is required to provide in the input directory, 10X Genomics post-CellRanger filtered sample contig output files. Meta file should contain file, sample id, batch, group, data type, and cell type (i.e. TCR or BCR contig files) information for each sample. For multimodal analysis with scRNA-Seq data, the corresponding scRNA-Seq data, *.h5* format post-Cellranger files, or Seurat object in *.RDS* format could also be provided in the input directory. A column in the meta file stating paired ids linking scRNA-Seq files with their corresponding immune contig files should be provided. Start the analysis with the following command:

scImmunePip(project_name = ‘My_scImmune’, input_dir = ‘/home/input/’, output_dir = ‘/home/output/’, pheno_file = ‘/home/input/meta.txt’)

#### Spatial Transcriptomics

For spatial transcriptomics, user is required to provide in the input directory, 10X Genomics post-Cellranger spatial transcriptomics *.h5* files and their corresponding spatial image files. Meta file should contain sufficient information to locate sample file, sample id and group information for each sample. If the user would like to conduct multimodal integrative analysis of each spatial data they provided with a corresponding scRNA-Seq and carry out cell type label transfer from these scRNA-Seq data, a sub-directory within the input directory containing these scRNA-Seq data should be provided. This sample should be named with the same prefix as their corresponding spatial samples. Command to start the analysis is as follows:

SpatialPip(project_name = ‘My_Spatial’, input_dir = ‘/home/input/’, output_dir = ‘/home/output/’, pheno_file = ‘/home/input/meta.txt’)

#### scCNV

For scCNV analysis, user is required to provide in the input directory, 10X Genomics post-Cellranger scCNV files. Meta file should contain sufficient information to locate sample file and sample id should be provided for each sample. Start the analysis with the following command:

scCNVPip(project_name = ‘My_scCNV’, input_dir = ‘/home/input/’, output_dir = ‘/home/output/’, pheno_file = ‘/home/input/meta.txt’)

#### CyTOF

For CyTOF analysis, user is required to provide in the input directory, post-live cells gating *.FCS* files. Meta file should contain file name, sample id, individual id, batch, and group information of each sample. Start the analysis with the following command:

CyTOFPip(project_name = ‘My_CyTOF’, input_dir = ‘/home/input/’, output_dir = ‘/home/output/’, pheno_file = ‘/home/input/meta.txt’)

#### Flow Cytometry Analysis Workflow

For flow cytometry analysis, user is required to provide in the input directory, raw *.FCS* files. Gating will be done automatically by Ursa. Meta file provided should contain file name, sample id, batch, and group information for each sample. One-liner command from R:

FlowPip(project_name = ‘My_Flow’, input_dir = ‘/home/input/’, output_dir = ‘/home/output/’, pheno_file = ‘/home/input/meta.txt’)

### Examples of Analysis Output

**Fig. 4.**
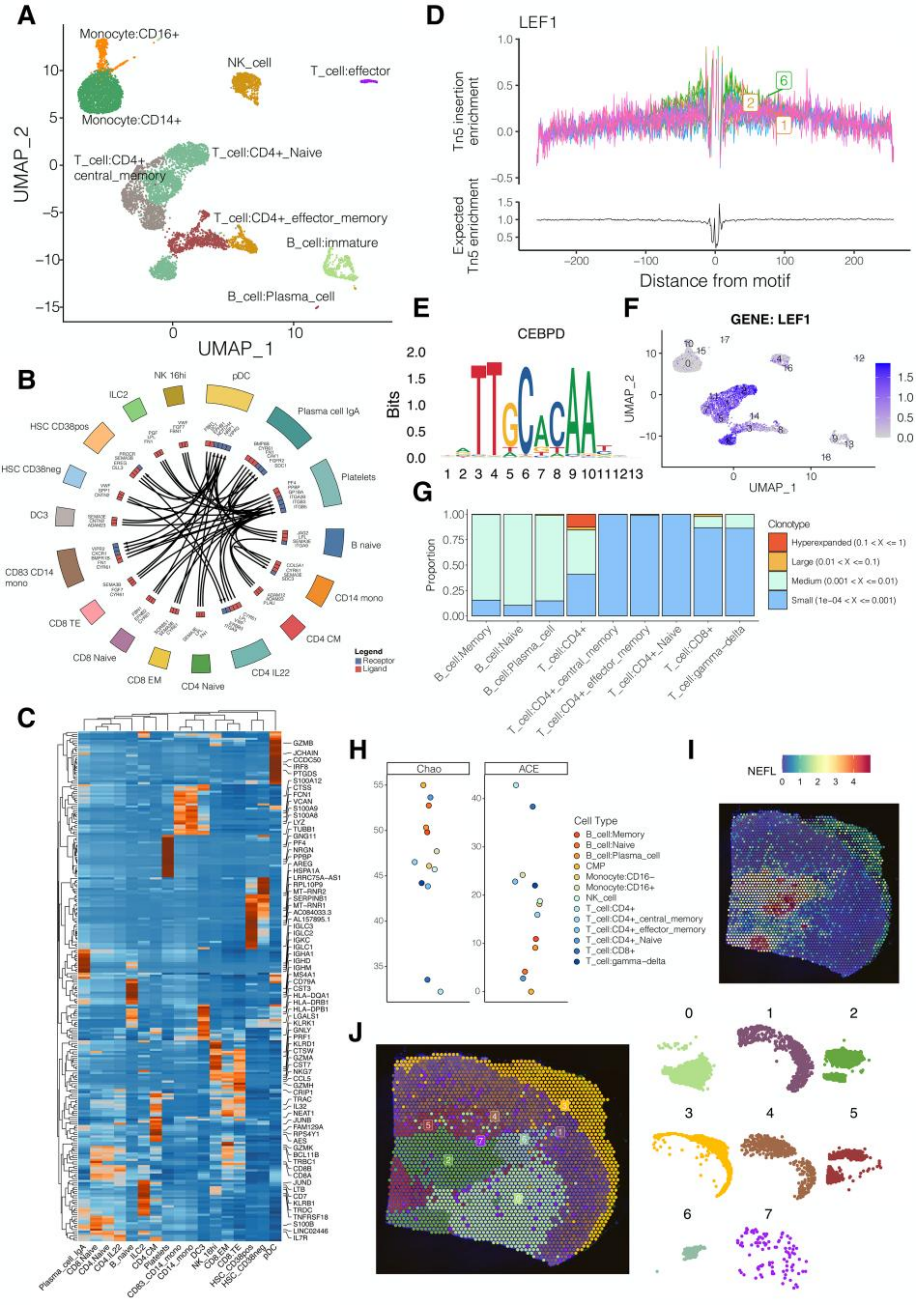
Visualization outputs from Ursa based on example data. (A) Post-dimensionally reduced UMAP plot of an integrated set of multimodal scImmune profiling PBMC samples, with clusters labeled by cell types. (B) Visualization of top receptor–ligand binding pairs in each predicted cell type of an integrated set of scRNA-Seq PBMC samples (supplementary table S2, Supplementary Material online), labled by cell types. Directions of the arrows indicate ligand-to-receptor binding patterns. (C) Heatmap of top DEGs of cell types of an integrated set of scRNA-Seq PBMC samples (supplementary table S2, Supplementary Material online). Top three DEGs in each cell type are labeled. (D) Visualization of a TF foot-printing analysis in a scATAC-Seq PBMC sample (supplementary table S2, Supplementary Material online). Tn5 insertion enrichment patterns of transcription factor LEF1 in each cluster (color representing clusters) are shown. (E) Visualization of position weight matrix of another transcription factor CEBPD after a motif analysis for a scATAC-Seq PBMC sample (supplementary table S2, Supplementary Material online). *x* axis shows the positioning of the motif, with *y* axis indicating the positional frequency of nucleotides. (F) Inferred gene expression of LEF1 of a scATAC-Seq PBMC sample (supplementary table S2, Supplementary Material online) across clusters. Labeled numbers indicate cluster numbers. (G) Clonal homeostasis of an integrated set of multimodal scImmune profiling PBMC samples (supplementary table S2, Supplementary Material online). *x* axis showing the cell types predicted from the scRNA-Seq samples, and *y* axis showing the proportion of each clonotype group present in samples. (H) Estimated clonal diversity of cell types of an integrated set of multimodal scImmune profiling PBMC samples (supplementary table S2, Supplementary Material online). *x* axis showing cell types and *y* axis displaying diversity scores for the methods. Higher the score indicates higher diversity in the cell types. (I) Spatial expression of neurofilament light chain (Nefl) in a spatial transcriptomics spinal cord sample (supplementary table S2, Supplementary Material online). Expressions of NEFL were mapped onto the histology image of the sample. (J) Spatial clusters mapped onto the histology image of a spatial transcriptomics spinal cord sample (supplementary table S2, Supplementary Material online).

To provide a better idea of the output of Ursa, here we provided some examples of main output visualizations from various omics using some example datasets (supplementary table S3, Supplementary Material online, Fig. 4). Additional visualization output can be found in the examples provided on the Github repository. For scRNA-Seq or multimodal scImmune profiling data, users will obtain a post-dimensionally reduced UMAP of predicted cell types by Ursa (Fig. 4a). Other post-dimensionally reduction visualizations include PCA and *t-*SNE plots. For cell–cell communication analysis, the visualization of top most significant receptor–ligand interaction pairs in each predicted cell type will be provided by Ursa to the user (Fig. 4b). To visualize top differentially expressed genes (DEGs) of cell types, a heatmap will be provided with top 3 DEGs in each cell type been labeled (Fig. 4c). Similarly, heatmap for DEGs of clusters will also be provided. For scATAC-Seq, visualization of transcription factor (TF) foot-printing analysis of each clusters will be provided (Fig. 4d). In this example, Tn5 insertion enrichment patterns of transcription factor LEF1 in each cluster (color representing clusters) is shown (Fig. 4d). In addition, enriched motif sequences in terms of position weight matrix for motif analysis in scATAC-Seq workflow will be visualized (Fig. 4e). As Ursa also provide gene expression inference for scATAC-Seq data based on their peak information, inferred gene expression of enriched TF will be visualized (Fig. 4f). For scImmune profiling multimodal data, clonal homeostasis and clonal diversities of cell types using different diversity methods will be visualized using stacked bar and dot plots (Fig. 4g and h). Another example is the spatial transcriptomics. Top spatial DEGs, for instance, the spatial gene expression of neurofilament light chain (Nefl) in a spinal cord sample (supplementary table S3, Supplementary Material online), will be mapped onto the histology image of the sample (Fig. 4i) to provide the user with a spatial sense of how the expression of the DEGs interspersed within the specimen. Post-dimensional and clustering results of the spatial expression data will also be visualized. To better facilitate clustering visualization, the spatial orientation of each cluster will be separately plotted (Fig. 4j).

### Comparison of Default Settings With Varying Thresholds Across Omics

**Fig. 5.**
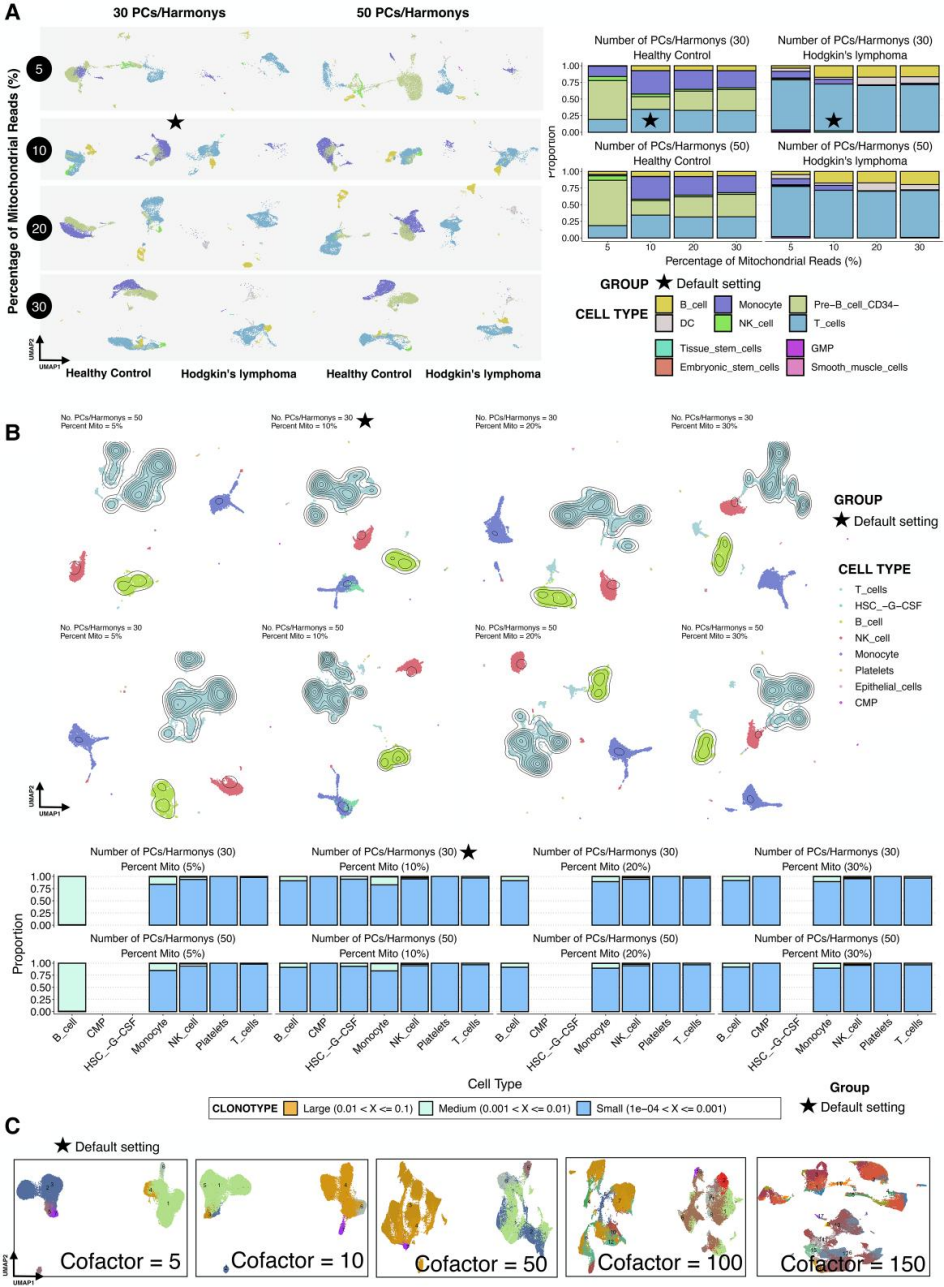
Comparison of default settings with varying thresholds in scRNA-Seq, scImmune profiling, and CyTOF. (A) UMAP representations showing the result of dimension reduction, grouped by cell types, across different parameter settings for scRNA-Seq data. Stacked bar plots showing the proportion of cell types across metrics in both healthy and diseased groups. Results from the default settings of Ursa are marked with a star symbol. (B) UMAP representations showing the result of dimension reduction, grouped by cell types, across different parameter settings for scImmune profiling data. Contours on each visualization depict the clonal frequency in each cell cluster. The higher the frequency, the higher the number of rings observed in each cluster of contours. Stacked bar plots showing the clonal homeostasis of each cell types across different metrics settings. Results from the default settings of Ursa are marked with a star symbol. (C) UMAP representations showing the result of dimension reduction, grouped by cell clusters, across a range of cofactor values for CyTOF data. Result from the default settings of Ursa is marked with a star symbol.

**Fig. 6.**
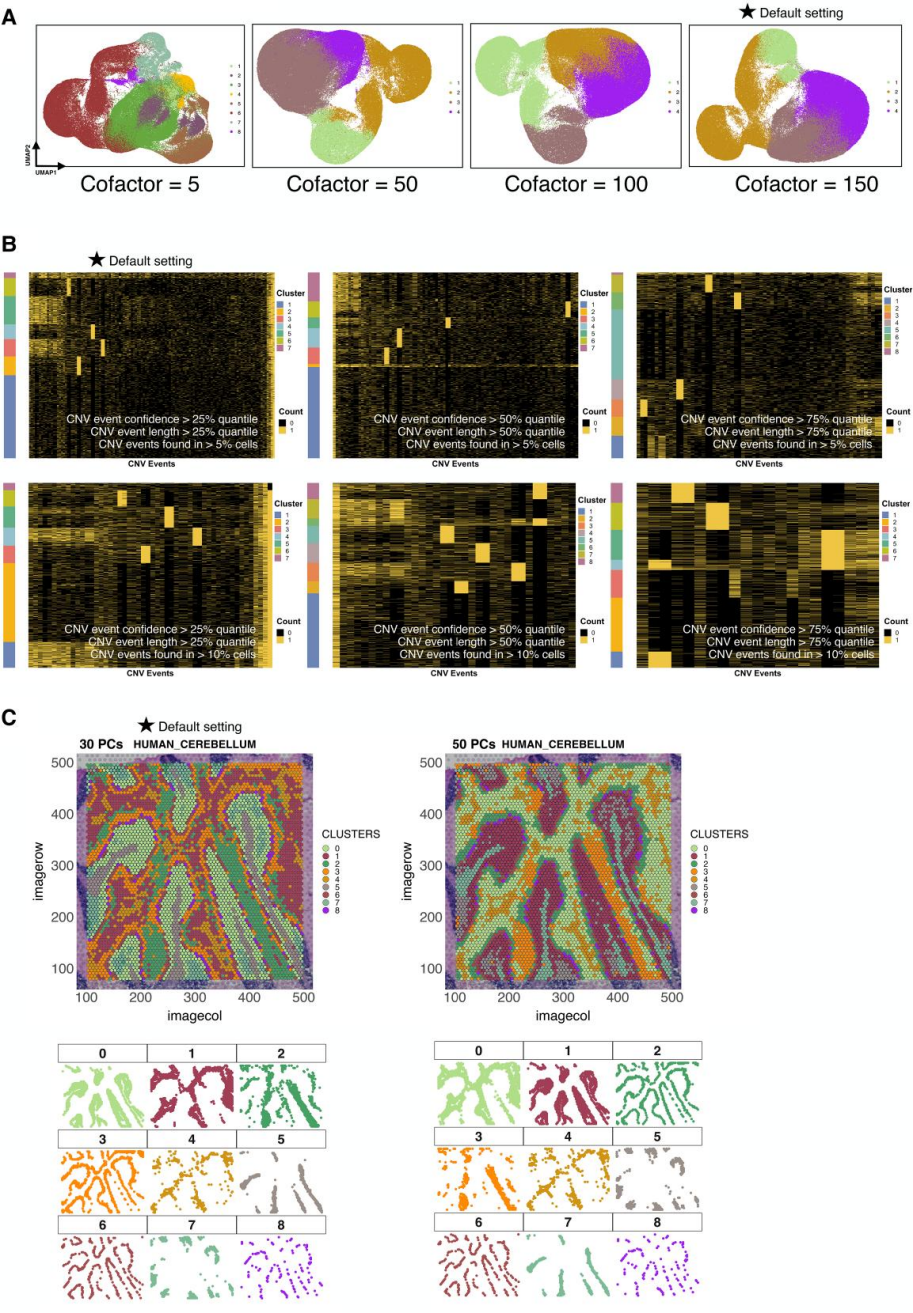
Comparison of default settings with varying thresholds in flow cytometry, scCNV, and spatial transcriptomics. (A) UMAP representations showing the result of dimension reduction, colored by cell clusters, across a range of cofactor values for flow cytometry data. Result from the default settings of Ursa is marked with a star symbol. (B) Heatmap showing the top CNV events, clustered by binary event counts, across different parameter settings for scImmune profiling data. Result from the default settings of Ursa is marked with a star symbol. (C) Result of dimension reduction, colored by cell type clusters, mapped onto the histology image of the brain sample in 2 different parameter settings for spatial transcriptomics data. Result from the default settings of Ursa is marked with a star symbol.

We have conducted comparisons of results for the default settings of Ursa in various omics against variations of these settings in important parameters such as the number of PCs or Harmonys used for dimension reduction and results are shown in Figs. 5 and 6.

#### scRNA-Seq Analysis

Four samples from 10X Genomics, with healthy peripheral blood mononuclear cells (PBMCs) from 2 healthy control samples and lymph node tumors from 2 Hodgkin's Lymphoma samples were used to assess how varying parameters, notably mitochondrial read percentages (commonly found in literature as 5%, 10%, 20%, 30%) and the count of PCs or Harmonys used (either 30 or 50), influenced the analytical outcomes. As shown in Fig. 5a, the distance between the cell types and the proportion of cell types in both the healthy and the Hodgkin's Lymphoma samples these variations are similar, especially for results of 10%, 20%, and 30% mitochondrial filtering with both 30 and 50 PCs or Harmonys. Higher proportion of the Monocyte is observed in the 10% mitochondrial cutoff as compared to the 20% and 30% cutoff for both 30 and 50 PC/Harmony settings in both healthy and diseased groups. Comparing across the results from these settings, the distributions of cell types in both normal and diseased groups for the 5% mitochondrial cutoff in both 30 and 50 PC/Harmony settings are very different as compared to all other mitochondrial cutoffs in both PCs/Harmony settings. Dendritic cells (DC) and B_cell seemed to being the major cells affected by the mitochondrial filtering, especially at 5% and 10% mitochondrial cutoffs in the diseased group.

The number of PCs or Harmonys did not seem to affect the proportions of cell types in both healthy and diseased groups when comparing 30 with 50 PCs or Harmonys within the same mitochondrial cutoff (Fig. 5a). However, the distance between cell types, especially Monocyte and Pre-B_cell_CD34-, are slightly more distant in the 50 PCs/Harmony setting as compared to the 30 PCs/Harmonys at 30% mitochondrial cutoff.

#### scImmune Profiling

For multimodal scImmune profiling combined with scRNA-Seq analysis, we employed the same parameter variations (specifically, mitochondrial percentages and PC/Harmony counts) on 2 healthy PBMC samples. Two multimodal healthy PBMCs samples with both TCR and BCR libraries sequenced were used in this analysis. As shown in Fig. 5b, clonotype frequencies shown in the contours of UMAP visualizations (i.e. the higher number of rings in the contours, the higher the clonal frequency in the center of each contour) are remarkably consistent across various settings, particularly for T_cells, B_cell, and NK_cell, and Monocyte. A noticeable uptick in T_cell frequencies was seen as the mitochondrial cutoff percentage increased.

Stratifying the clonal frequencies into different categories as shown in the stacked bar plots in Fig. 5b, some of the small proportion cell types, such as CMP and HSC_-G-CSF, disappeared at 5%, 20%, and 30% mitochondrial cutoff, while the others, except for the B_cell in 5% mitochondrial cutoff, demonstrated similar clonal homeostasis patterns across varying mitochondrial cutoff and number of PC/Harmony settings. The B_cells demonstrated medium expansion almost for all cells in the 5% mitochondrial category and has dropped to the small expansion category after loosening the mitochondrial cutoffs to 10%, 20%, and 30%. Of all, the default setting used by Ursa demonstrated a complete set of cell types found across the settings (Fig. 5b).

#### CyTOF

We evaluated the cofactor value used for data standardization using 5 pairs of healthy PBMC samples (Bodenmiller et al. 2012; Nowicka et al. 2017). Of each pair, one was naïve while the other was stimulated using B/Fc cell receptor cross-linker (BCR-XL) (Bodenmiller et al. 2012; Nowicka et al. 2017). As shown in Fig. 5c, at the default setting of cofactor = 5, dimension reduction and clustering steps demonstrated distinct and clean clustering of cells. However, as the value of cofactor increases, the clarity of these clusters diminished, and became absurd and notably unclear at a cofactor = 150 (Fig. 5c).

#### Flow Cytometry

Similarly, we assessed the cofactor values used for flow cytometry data using 10 activated samples sourced from CytoExploreRData (Hammill 2020). As shown in Fig. 6a, a default cofactor of 150 resulted in sharp and distinct cell clustering patterns, and the performance echoed at a cofactor of 100. At a setting of 50, slightly noisy clusters were observed. When reduced to a cofactor of 5, the results displayed a higher number of clusters, with several of the clusters overlapping or blending with others (Fig. 6a).

#### scCNV

Using 2 breast cancer samples sourced from 10X Genomics, we assessed the top CNV events (binary count) based on a range of filtering criteria, as depicted in Fig. 6b. The default setting revealed 4 clear clusters across chromosomes. Yet, as filtering criteria became stricter, the number of clear clusters rose to approximately 6. This stricter filtering, especially when raising the threshold of retaining CNV events from those present in more than 5% of cells to 10%, introduced greater noise between the clusters (Fig. 6b).

#### Spatial Transcriptomics

Employing a brain cerebellum sample from 10X Genomics, we evaluated the impact of different numbers of PCs on dimension reduction outcomes. Figure 6c shows consistent clustering patterns regardless of the number of PCs applied, echoing findings from scRNA-Seq test outcomes when adjusting PC settings while holding other variables intact.

#### scATAC-Seq

ScATAC-Seq data is known to have a high degree of sparsity compared to other single-cell assays, making it more susceptible to noise and artifacts with parameter variations. Given these considerations, we opted to refrain from testing variable parameters on the scATAC-Seq dataset in this analysis and stick to the use of recommended quality control settings by Signac (Stuart et al. 2021).

## Discussion

In our endeavor to simplify multiomics analysis, we present Ursa: a comprehensive solution encapsulated in a single R package. It delivers high-quality, high-resolution, publication-ready visualizations with minimal coding, seamlessly integrating popular tools for an all-in-1 analysis experience. Ursa bridges the gap between various analytic tools, addressing potential data or format compatibility issues that might arise during the transition from 1 analysis tool to another. Every parameter and cutoff in Ursa are meticulously curated, simulating the expertise of a bioinformatician while retaining user adjustability. Ursa consolidates the strengths of well-established packages, including Seurat (Hao et al. 2021), Signac (Stuart et al. 2021), Monocle3 (Trapnell et al. 2014; Qiu et al. 2017; Cao et al. 2019), Harmony (Korsunsky et al. 2019), and more, always adhering to the recommended or default settings stipulated by these software solutions.

However, every innovation comes with its set of challenges and limitations. As Ursa amalgamates numerous analytic tools, significant updates to any of these tools necessitate immediate assessment on our end to ensure the seamless functionality of Ursa. Additionally, while some users might lean toward a UI version of the software, we currently do not provide this feature.

It is important to note that, even though we have set a series of default recommended settings for running the omics analysis based on literature and dependency software, it is essential for users to meticulously document the selected thresholds in their methodologies to ensure transparency and reproducibility. While Ursa is primarily designed for individuals who might not possess advanced computational skills, we still encourage these users to seek guidance from pertinent academic literature when determining certain statistical parameter thresholds, such as the BH FDR threshold essential for multiple testing corrections.

With the growing emphasis on multiomics studies, we anticipate the role of Ursa to become indispensable, offering a streamlined solution to diverse omics datasets. We are confident that Ursa will bring substantial benefits to the broader research community, especially for bench scientists and those less acquainted with computational nuances. Looking ahead, our vision for Ursa includes the incorporation of additional bulk omics techniques and a wider array of single-cell omics options, thereby curating a holistic suite of analysis workflows for contemporary omics research.

## Materials and Methods

Ursa analysis workflow for each omics has been summarized in Fig. 3. Below, we provide R commands to run the workflow of each omics and detailed descriptions of the methods used by each of the omics workflows in Ursa.

### scRNA-Seq Analysis Workflow

#### Quality control (QC) assessment

For each sample in the project, a series of QC metrics are calculated using Seurat (Hao et al. 2021) (Fig. 3). The percentage of mitochondrial genes expressed in each cell will be calculated to discover damaged cells or artifacts present in each sample. The number of genes and RNA molecules detected per cell is calculated simultaneously. Cell cycle effect is assessed via *CellCycleScoring* function in Seurat. Samples are normalized with *NormalizeData* and scaled using *ScaleData* functions. PCA and UMAP are run, using *RunPCA* and *RunUMAP*, respectively, based on a list of cell cycle markers (S and G2M phase markers) and highly variable genes, to compare and contrast the effect of cell cycle markers on the data. If there is an obvious cell cycle heterogeneity observed in the samples, depending on the biological question of the project, the user could choose to run the project with the removal of cell cycle effect on the data, using additional parameter *cc_regression* in the command (by default, cell cycle effect will not be removed, i.e. *cc_regression = 0*). Two options are available to the user, including 2 phases (*cc_regression = 1*), and phase difference cell cycle regressions (*cc_regression = 2*). The former will mitigate the effect introduced by all cell cycle phases and the latter will remove only the difference between G2M and S phases and is done using *ScaleData* function in Seurat. The pre-filtering results is visualized and presented to the user. Filtering is then carried out for each sample, cells with the number of genes expressed between 200 (exclusive) and 25,000 (inclusive), and less than 5% of mitochondrial genes expressed (exclusive) is retained by default, and is currently catered for 10X Genomics (Weisenfeld et al. 2017; Zheng et al. 2017). This step removes possible empty droplets, doublets, artifacts, and damaged cells present in the sample as a result of experimental procedures.

#### Dimension reduction and clustering

For each sample, PCA, UMAP, and *t*-SNE projections are calculated using *RunPCA*, *RunUMAP*, and *RunTSNE* in Seurat. Clustering is done using *FindClusters* in Seurat with a resolution of 0.8. Clustering results will be shown in 2D and 3D interactive UMAP and *t*-SNE plots.

#### Integrative analysis

For a project with multiple samples submitted, integration of samples is carried out. An example of such a project is a comparison between healthy and diseased samples. Group information should be provided in the metadata submitted. Depending on the choice of the integration method, the integration is done using Seurat or Harmony (Korsunsky et al. 2019). If Seurat integration is selected, a more stringent integration is performed by considering each sample as a batch. This is beneficial for projects with high intra-batch variations. If the Harmony method is selected, the integration is done batch-wise, based on the batch information in the provided meta file. In terms of time complexity, Harmony will perform at a faster pace as the sample size increases. Post-integration dimension reduction on UMAP and *t*-SNE will be performed based on PCA embeddings for Seurat integration, and harmony embeddings for Harmony integration. Clustering is done using *FindClusters* in Seurat with a resolution of 0.8. Clustering results will be provided in the forms of 2D and 3D interactive UMAP and *t*-SNE plots in html format.

#### DEGs Discovery

DE analysis is performed for each cluster using *FindAllMarkers* in Seurat. To aid cell-type annotation, cell type prediction process will be carried out using SingleR (Aran et al. 2019) based on the clustering result, and Human Primary Cell Atlas (Mabbott et al. 2013) is used as the annotation reference. Manual annotation check based on DEGs is encouraged. For a project with multiple groups, group-wise comparison within each cluster is performed using *FindAllMarkers* function from Seurat.

#### Pseudotime trajectory analysis

Monocle3 is used for pseudotime analysis (Cao et al. 2019). The principle graph is first learned using *learn_graph* and to maximize flexibility in the result, each cluster is used as a starting node for pseudotime computation, and each of these results will be made available to the user. This allows the user to choose the pseudotime trajectory result based on their biological questions.

#### Enrichment analysis

For each cluster of each sample, 500 top DEGs, ranked by decreasing average log-fold change, are used for enrichment analysis of disease-gene associations based on DisGeNET database (Piñero et al. 2016), using *enrichDGN* in DOSE (Yu et al. 2014). Enrichment disease terms below *P* < 0.05 and *q* < 0.05 are retained. GSEA is also performed on the DEGs of each cluster using *gseNCG* in DOSE.

#### Receptor–ligand binding analysis

For each cluster and predicted cell type, cell–cell communication analysis is carried out using *celltalk* in celltalker (Cillo et al. 2020), to identify receptor–ligand interactions in each cluster or cell type.

### scATAC-Seq Analysis Workflow

#### QC Assessment

Workflow is catered for 10X Genomics post-Cellranger output files. The nucleosome banding pattern is computed and visualized using Signac (Stuart et al. 2021), and this is done for each cell. The transcription start site (TSS) enrichment score is calculated to examine the enrichment level in TSS regions. The number of fragments in peaks is used to examine cellular sequencing depth and complexities. Too low or high peak counts are indications of low sequencing depths or doublets. Another QC check is the identification of cells with a high proportion of reads mapped to blacklist regions. These regions, identified by ENCODE (Dunham et al. 2012), are a list of signal-artifact regions with erroneous signals. The ratio of reads in each cell mapped to blacklist regions compared to overall reads present in the cell is calculated. Based on the QC metrics, for each sample, cells with (i) number of fragments in peaks > 3,000 and <20,000; (ii) nucleosome signal < 4; (iii) TSS enrichment score > 2; (iv) blacklist ratio < 0.05; and (v) percentage of reads at the peak regions > 15% are retained. This filtering process removes cells with high percentage of reads mapping to blacklist regions, low-quality cells with low sequencing depths, and potentially doublets with extreme high number of fragments in peaks.

#### Dimension Reduction and Clustering

Normalization is performed using *RunTFIDF* and *RunSVD* in Signac. The first component of LSI often captures technical variation than biological and thus is dropped (Stuart et al. 2021). For a project with multiple samples, samples will be integrated using Seurat after sample normalization. Dimension reduction is carried out using *RunUMAP* and *RunTSNE* in Seurat to generate UMAP and *t*-SNE embeddings. After dimension reduction, the clustering step is carried out based on the 2nd to 30th LSI components using *FindClusters* function in Seurat.

#### DE Peaks Discovery

Differentially accessibility analysis is performed for each cluster on each peak using logistic regression in Signac. The number of peaks is used as a latent variable to reduce the sequencing depth effect. For each comparison, peaks that cannot be detected at a minimum of 0.05 in proportion in either of the 2 groups of cells are discarded.

#### Inferring Gene Expression

To relate the promoter accessibility of each gene to its gene expression, using Signac, the number of fragments mapped to the 2 kb upstream region of every gene is tabulated and the number of fragments at the region is used as an activity score for each gene in each cell. The gene activity matrix is normalized and scaled using Seurat. Based on the clustering results of the scATAC-Seq data, DEGs will be computed using *FindAllMarkers* in Seurat. For each peak, GC content, accessibility, and peak length are computed. Correlation analysis is carried out using gene activity of every top gene derived from the DE analysis with all peaks nearby the gene.

#### Motif Analysis and TF Foot-Printing

DNA sequence motif analysis is subsequently carried out using Signac. A collection of motif positions is first queried from the JASPAR database (Fornes et al. 2020) and mapped to the peaks. Motif enrichment analysis is carried out on the DEGs to find enriched motifs in these genes and their position weighted matrices. Foot-printing information for motifs is computed using *Footprint* function in Signac.

### scImmune Profiling Analysis Workflow

#### Clonal Assessment

Workflow is catered for 10X Genomics 5′ V(D)J protocol Cellranger output files. For each repertoire of every sample, the number of unique clonotypes, distribution of clonal abundance, and CDR3 sequence length distribution are computed using scRepertoire (Borcherding et al. 2020). Through binning, repertoires occupancies is assessed in various ways, first is to rank the repertoires by their proportions, and they is then split into bins, with the first bin containing the top 1 to 10 clonotypes, the next bin containing the top 11 to 100 clonotypes, and so on. Given that immune repertoires are diverse in nature due to random recombination of VDJ sequences, this way of representation gives more weight to the top clonotypes in terms of the visualization process (similar to log scale transformation). The next is to examine the rare clonal proportions, the top bin consists of the rare clonotypes with single cells, and the second is clonotypes with 2 to 3 cells, and so on. The concept is similar here as compared to the first way of binning the visualization process. Clonal space homeostasis will also be assessed, through binning the clonotypes with different proportions into rare (0 < X ≤ 1e^−04^), small (1e^−04^ < X ≤ 0.001), medium (0.001 < X ≤ 0.01), large (0.01 < X ≤ 0.1), and hyper-expanded (0.1 < X ≤ 1) bins/groups.

#### Shared Clonotypes Analysis

For multiple samples project, repertoire similarity assessments are conducted, to examine the level of overall, as well as top clonotypes sharing between samples, and this is indeed very useful for different biological aims. To measure the degree of similarities between repertoires, both the Morisita overlap index and a direct comparison of the number of overlapping clonotypes are computed and displayed.

#### VDJC Gene Usage

This step is to dissect differential gene usage across repertoires (Borcherding et al. 2020), to observe similarities, and also, to examine what proportion of combination of genes contributes to the repertoire diversities. The correlation matrix and usage by JS-divergence for each gene will also be calculated. Hierarchical clustering of the repertoires is done for each gene, based on the gene usage, using the cosine similarity procedure. Clonal diversity will also be estimated (Fig. 3).

#### Multimodal Integration

For multimodal samples consisting of immune repertoires and their complementary scRNA-Seq samples, scRNA-Seq sample files of the repertoires are read in, and filtered before the multimodal integrative analysis. Cells with <200 or >6,000 genes expressed are discarded. Post-filtering data is then normalized and scaled using Seurat. This is followed by dimension reduction to PCA and UMAP using Seurat. If multiple samples are supplied, an integrative analysis is done for the scRNA-Seq samples. Depending on the integration method chosen by the user, the same procedure for Seurat or Harmony integration, post-integration dimension reduction, and clustering are conducted as described in the scRNA-Seq workflow. Automated cell annotation is carried out to predict the cell-type identity of each cluster using SingleR with Human Primary Cell Atlas as the annotation reference.

#### Multimodal Mapping

scRepertoire is used in this analysis. Immune repertoires are mapped to their respective scRNA-Seq samples and based on their unique cell barcodes, clonotype calls on VJDC genes and the CDR3 nucleotide sequence is subsequently mapped. The proportion of clonal expansion groups will be calculated for each predicted cell type, for an assessment of the proportion of expansion in each cell type group. Function *clonalDiversity* is used to compute diversities of predicted cell types in multimodal data. To assess the network interaction of clonotypes shared between cell types, a clonal network is computed and reflected onto UMAP embeddings.

### Spatial Transcriptomics Analysis Workflow

#### Data Processing

Workflow is catered for 10X Genomics post-Cellranger output files. Using Seurat, for each sample, the number of RNA molecule counts for each spot is calculated. Normalization for technical variations is done using *sctransform* in Seurat. For a project with multiple samples, downstream analysis is carried out separately for each sample.

#### Dimension Reduction and Clustering

As spatial transcriptomics are essentially transcriptomics data, therefore similar procedures compared to scRNA-Seq workflow follow. For each sample, PCA, UMAP, and *t*-SNE projections are calculated using *RunPCA*, *RunUMAP*, and *RunTSNE* in Seurat. Clustering is done using *FindClusters* in Seurat with a resolution of 0.8.

#### DEGs Discovery

DE analysis is performed for each cluster using *FindAllMarkers* in Seurat. To aid cell-type annotation, cell type prediction process will be carried out using SingleR with Human Primary Cell Atlas as the annotation reference. Manual cell-type annotation based on the provided DEGs is also encouraged.

#### Multimodal Integrative Analysis

For a project with both spatial transcriptomics and scRNA-Seq samples provided, the scRNA-Seq samples will be processed similar to the scRNA-Seq workflow. Cells with <200 or >6,000 genes expressed in 1 cell are discarded. Cutoff is currently catered for 10X Genomics output. Data is normalized using the same *sctransformed* method as the spatial samples to prepare for integration. PCA is run followed by UMAP, which uses the first 30 PCA embeddings. Clustering is carried out using *FindClusters*. Automated cell annotation is carried out to predict the cell-type identity of each cluster and Seurat integration is performed by first identifying a set of transfer anchor cells between the spatial data and the scRNA-Seq data, followed by a transfer of the predicted cell type labels in the scRNA-Seq data onto the spatial sample.

### scCNV Analysis Workflow

#### QC Assessment

Workflow is catered for 10X Genomics post-Cellranger output files. For each sample, cells with low ploidy confidence scores ≤ 2 or outlier cells that significantly deviated from the fitted distribution are discarded (Zheng et al. 2017). Diploid cells with CNV event confidence ≤ 100 and non-diploid cells with event confidence ≤ 50 are also discarded. Based on the CNV profiles of each cell, hierarchical clustering is carried out to group cells with similar ploidy levels, to give an overview of the sample ploidy profile. The mean ploidy of each sample, based on the copy number profile of each CNV event, is computed.

#### Analysis of Non-diploid Cells

Non-diploidy cells are selected for downstream analysis. Non-diploid cells with CNV event size in the mappable regions > 2000 kb with confidence > 50 are retained. CNV events present in less than 5% of all cells in the sample are removed. The final set of CNV events is binarized and clustering is done using the Discriminant Analysis of Principal Components (Jombart et al. 2010). Successive k-means is run using PCA components, and the goodness of fit measure using the Bayesian information criterion (BIC) (Schwarz 1978) is computed. Based on the BIC information, the number of clusters is automatically determined. Dimension reduction using UMAP is performed on the binarized CNV events. Hierarchical clustering is carried out on the binarized non-diploid matrix, to observe any obvious CNV clusters in each cell cluster. The top 50 CNV events in the non-diploid cells are selected for hierarchical clustering, based on their CNV ploidy information at each chromosome position. For a multiple samples project, the proportion of cluster occupancy of the non-diploid cells for each sample is assessed and plotted and a phylogenetic tree is plotted by comparing and clustering the clusters based on their median copy number.

### CyTOF Analysis Workflow

#### QC and Data Processing

FCS format files post-live cell gating is read in and scaling is performed by arcsine transformation on the expression matrix of each sample with a cofactor of 5. If batch information is provided, batch removal is carried out to regress out the batch variable using linear regression. MDS embeddings are computed for each sample and used for sample-wise distance check, to observe intra- and inter-group heterogeneity. Non-redundancy scores (NRS) (Nowicka et al. 2019) are computed for each marker in each sample to identify highly variable markers across the samples.

#### Dimension Reduction and Clustering

FlowSOM (Van Gassen et al. 2015; Nowicka et al. 2019) is used to first carry out the first level of dimension reduction and clustering on the batch-corrected samples, by reducing the cell dimension to 100 dimensions. This forms a set of 100 meta-clusters (or SOM clusters) each containing a set of highly similar cells. Secondary clustering is then carried out using the consensus clustering (CC) (Nowicka et al. 2019) method based on the meta-clusters. Elbow method is used to determine the final cluster number based on the CC output. Meta-clusters are dimensionally reduced by UMAP and *t*-SNE and all cells are dimensionally reduced using UMAP.

#### Phenotypic Discoveries

Post-clustering, the median expression of the markers for each meta cluster or secondary cluster, is computed. Cell identities for each cluster and each meta cluster are predicted by computing the DE markers of each cluster. If the median expression of a marker in a cluster has a fold change > 1.25 as compared to the median expression of the marker expressing in all cells, this cluster will be phenotypic ally with this marker, together with other DE markers. The list of a final set of markers, for example, CD3 + CD4 + for cluster 1, will be output as the predicted phenotype for the cluster.

### Flow Cytometry Analysis Workflow

#### QC and Gating Strategies

Raw FCS format files are read in and scaling is performed by arcsine transform the expression matrix of each sample with a cofactor of 150. Automated gating is carried out to remove debris or doublets. The gating strategies for selecting live cells are as follows: (i) forward scatter area (FSC-A) versus side scatter area (SSC-A) gating; followed by (ii) FSC-A versus forward scatter height (FSC-H) gating; (iii) side scatter height (SSC-H) versus side scatter width (SSC-W) gating; and (iv) forward scatter width (FSC-W) versus forward scatter height (FSC-H) gating. MDS embeddings are computed for each sample and used for sample-wise distance check. NRS values are computed for each marker in each sample to identify highly variable markers across the samples.

#### Dimension Reduction and Clustering

Similar to CyTOF workflow, flowSOM is used to first carry out the first level of dimension reduction and clustering on the batch-corrected samples, by reducing the cell dimension to 100 dimensions. This forms a set of 100 meta-clusters (or SOM clusters) each containing a set of highly similar cells. Secondary clustering is then carried out using the CC (Nowicka et al. 2019) method based on the meta-clusters. Elbow method is used to determine the final cluster number based on the CC output. All cells are dimensionally reduced using UMAP. Median expression of each marker for each secondary cluster is computed to allow user to identify cell phenotype for the cluster.

## Supplementary Material

Supplementary material is available at *Molecular Biology and Evolution* online.

## Supplementary Material

msad267_Supplementary_Data

## Data Availability

The package and its source codes, as well as examples files and meta files, are available at: https://github.com/singlecellomics/ursa.
